# Enhancing sentiment and intent analysis in public health via fine-tuned Large Language Models on tobacco and e-cigarette-related tweets

**DOI:** 10.3389/fdata.2024.1501154

**Published:** 2024-11-28

**Authors:** Sherif Elmitwalli, John Mehegan, Allen Gallagher, Raouf Alebshehy

**Affiliations:** Tobacco Control Research Group, Department for Health, University of Bath, Bath, United Kingdom

**Keywords:** social media analysis, sentiment analysis (SA), intent classification, Large Language Models (LLMs), public health, domain adaptation, tobacco, e-cigarette

## Abstract

**Background:**

Accurate sentiment analysis and intent categorization of tobacco and e-cigarette-related social media content are critical for public health research, yet they necessitate specialized natural language processing approaches.

**Objective:**

To compare pre-trained and fine-tuned Flan-T5 models for intent classification and sentiment analysis of tobacco and e-cigarette tweets, demonstrating the effectiveness of pre-training a lightweight large language model for domain specific tasks.

**Methods:**

Three Flan-T5 classification models were developed: (1) tobacco intent, (2) e-cigarette intent, and (3) sentiment analysis. Domain-specific datasets with tobacco and e-cigarette tweets were created using GPT-4 and validated by tobacco control specialists using a rigorous evaluation process. A standardized rubric and consensus mechanism involving domain specialists ensured high-quality datasets. The Flan-T5 Large Language Models were fine-tuned using Low-Rank Adaptation and evaluated against pre-trained baselines on the datasets using accuracy performance metrics. To further assess model generalizability and robustness, the fine-tuned models were evaluated on real-world tweets collected around the COP9 event.

**Results:**

In every task, fine-tuned models performed much better than pre-trained models. Compared to the pre-trained model's accuracy of 0.33, the fine-tuned model achieved an overall accuracy of 0.91 for tobacco intent classification. The fine-tuned model achieved an accuracy of 0.93 for e-cigarette intent, which is higher than the accuracy of 0.36 for the pre-trained model. The fine-tuned model significantly outperformed the pre-trained model's accuracy of 0.65 in sentiment analysis, achieving an accuracy of 0.94 for sentiments.

**Conclusion:**

The effectiveness of lightweight Flan-T5 models in analyzing tweets associated with tobacco and e-cigarette is significantly improved by domain-specific fine-tuning, providing highly accurate instruments for tracking public conversation on tobacco and e-cigarette. The involvement of domain specialists in dataset validation ensured that the generated content accurately represented real-world discussions, thereby enhancing the quality and reliability of the results. Research on tobacco control and the formulation of public policy could be informed by these findings.

## Introduction

In the digital age, social media platforms have emerged as vital sources of popular opinion and mood. The large volume of user-generated information on these platforms provides scholars, policymakers, and organizations with unprecedented access to real-time expressions of public opinion on a wide range of issues. Sentiment analysis (SA), which uses powerful natural language processing techniques, has emerged as a critical tool for extracting relevant insights from this vast amount of data (Rodríguez-Ibánez et al., 2023). Sentiment analysis can be applied to millions of postings, comments, and tweets to provide a complete view of public opinion on a wide range of issues, allowing for a more nuanced understanding of social trends, consumer behavior, and public reactions to events, commodities, or laws.

SA has a varied role in social media. Unlike traditional techniques of public opinion measurement, such as polls or focus groups, social media SA enables continuous monitoring of large-scale, spontaneous interactions. This technique has numerous advantages: it may detect developing concerns before they spread, follow the change of public opinion over time, and assess the effectiveness of various actions or campaigns. For businesses, it gives information on customer happiness and brand perception. For policymakers, it provides an indication of public reaction to new policies. For scholars, it opens new opportunities to investigate human behavior and social dynamics on a large scale (Chakraborty et al., 2020).

SA of tobacco and e-cigarette-related social media material specifically creates opportunities to advance public health. As discussions regarding tobacco use, e-cigarettes, regulation, and health consequences become more prevalent online, studying these interactions can yield vital insights for tobacco control efforts and public health measures. This research is especially relevant given the quickly changing landscape of nicotine and tobacco products (Vassey et al., 2020).

SA can enable public health officials to assess the efficacy of anti-tobacco and anti- e-cigarette campaigns and change strategies accordingly and can help identify specific communities with strong pro-tobacco or pro- e-cigarette attitudes, allowing for more targeted interventions. It can also identify widespread misconceptions or disinformation spread on social media, allowing for timely and targeted instructional responses (Lee et al., 2023).

Public sentiment and discussion intent are two important lenses for understanding social media discourse. SA examines underlying tones toward topics, from negative to positive. However, sentiment alone does not reveal the purpose or goal behind online interactions. Intent classification complements SA by categorizing posts' main objectives, such as advocating for/against issues (Rizou et al., 2023). Discerning intent is especially important for public health monitoring, allowing insights into whether conversations aim to promote or deter behaviors. Together, sentiment and intent analysis offer a comprehensive view of digital conversations to inform research, policymaking, and interventions.

The introduction of Large Language Models (LLMs) has transformed natural language processing, providing new opportunities for sentiment and intent analysis. LLMs, which have been pre-trained on vast amounts of text data before being fine-tuned for downstream tasks, have achieved significant success in Natural language Processing (NLP). These pre-trained models, such as BERT, GPT-4, and T5, leverage transfer learning by first learning general language representations from unlabelled data, which they can then apply to new labeled datasets through fine-tuning to certain domains, proves their potential for assessing the frequently complex and context-dependent language used in social media posts on tobacco and e-cigarettes (Kukreja et al., 2024; Askarbekuly and Aničić, 2024). The promise of LLMs in this context stems from their ability to manage the informal language, irony, and cultural allusions found in social media conversation, which sometimes cause issues for simpler models. This is especially useful in the context of research on tobacco and e-cigarette social media messaging, where huge, annotated datasets are not always accessible.

The Flan-T5 model, a variation of the T5 model fine-tuned for a variety of applications, offers a promising technique for SA of tobacco and e-cigarette-related tweets. While not as new or large as models such as GPT-4 or Claude 3.5, Flan-T5 offers various advantages that make it suitable for this application (Kanakarajan and Sankarasubbu, 2023; Chung et al., 2024):

1. Smaller Flan-T5 versions are computationally efficient, making them ideal for fine-tuning and deployment on systems with limited resources. This is especially important when dealing with big twitter datasets.
2. Flan-T5's “instruction tuning” feature provides unambiguous instructions for SA and intent categorization in tweets, potentially enhancing performance.
3. Adaptability: Flan-T5's adaptability in text-to-text tasks makes it suitable for SA and intent categorization in our study.
4. Flan-T5 is ideal for fine-tuning domain-specific tasks, such as evaluating tobacco and e-cigarette-related tweets, because of its lower size and restricted datasets.
5. Flan-T5's open-source nature promotes openness and customization, enabling researchers to tailor the model to their own requirements.

Furthermore, Flan-T5's ability to fine-tune to specific domains makes it well-suited for sentiment and intent analysis of tobacco and e-cigarette-related tweets, which requires understanding subtle language particular to discussions of tobacco products.

The goal of this study is to use Flan-T5 to improve sentiment and intent categorization of tweets about tobacco and e-cigarettes. Our main aims are as follows:

1. Developing and evaluating three unique LLMs: (a) A model to categorize tweets into three intents related to tobacco products: pro-tobacco, anti-tobacco, and neutral. (b) A model to categorize tweets into three intents related to e-cigarettes (pro-e-cigarette, anti-e-cigarette, and neutral). (c) A methodology for categorizing tweets into three attitudes toward tobacco and e-cigarettes (negative, neutral, and positive).
2. Compare pre-trained and fine-tuned models to see how domain-specific fine-tuning affects accuracy and robustness.
3. Compare model performance variances across tobacco and e-cigarette-related content to better comprehend the unique discourse around each product category.
4. Evaluate how well these models capture sophisticated language and context-specific expressions in social media debates concerning tobacco and e-cigarettes.

This study proposes that fine-tuning the Flan-T5 model using carefully curated datasets of tobacco and e-cigarette-related tweets significantly enhances its performance in sentiment analysis and intent classification tasks compared to the pre-trained model. In addition to the synthetic data used for training, validation, and testing, real-world tweets around the COP9 event were employed for further evaluation, validating the fine-tuned models' generalizability to real-world contexts. We hypothesize that the fine-tuned models are better equipped to accurately differentiate subtle expressions of sentiment and intent, as well as identify neutral or factual statements related to tobacco and e-cigarettes.

Having demonstrated the potential benefits of employing sentiment and intent analysis in tobacco and e-cigarette-related social media content and the potential of LLMs in this domain, it is necessary to examine the existing research that informs our approach. What follows is a summary of major articles in SA, tobacco and e-cigarette-related social media research, and an overview of the use of LLMs in related fields, laying the groundwork for our comparative study of fine-tuned and pre-trained Flan-T5 models.

## Related work

The application of LLMs in sentiment analysis, intent classification, and public health research has seen significant advancements in recent years. This section highlights key studies and identifies gaps that our work aims to address.

In social media sentiment analysis, several advanced techniques have emerged. Bonifazi et al. proposed a scalable methodology for sentiment analysis on social platforms, showing the potential for handling large volumes of text data, although their work was not directly applied to systematic reviews (Bonifazi et al., 2023). Their approach is valuable for efficiently processing large datasets, which can inform public health research and systematic literature screening. This emphasis on scalability aligns with our focus on using domain-specific fine-tuning for large models in analyzing tobacco-related content.

Tobacco-related sentiment analysis has also shown promising results. Elmitwalli et al. (2024) demonstrated the effectiveness of topic prediction and sentiment analysis on tobacco-related tweets, achieving high accuracy using pre-trained deep learning models. Another study by Elmitwalli and Mehegan (2024) found that deep learning approaches, including models like BERT, outperform traditional machine learning methods for sentiment analysis in the context of tobacco control (Galimov et al., 2024). These findings establish the groundwork for our study, which takes the next step by fine-tuning LLMs specifically for domain-related tasks to enhance both performance and computational efficiency.

The introduction of BERT by Devlin et al. (2018) marked a significant milestone in natural language processing, as it allowed for better contextual understanding of text through pre-training on large corpora. However, subsequent research has shown that pre-trained models often experience performance drops when applied to domain-specific tasks without fine-tuning (Kocoń et al., 2023). To address this, recent studies have explored the effectiveness of fine-tuning models for specific domains, with results showing significant improvements in accuracy (Chung et al., 2022). Our study builds on this line of research by fine-tuning a LLM, Flan-T5, for the analysis of tobacco and e-cigarette-related tweets.

Beyond tobacco research, studies in other domains have explored the performance of both pre-trained and fine-tuned LLMs. Bhattarai et al. (2024) conducted a comparative study between Flan-T5 and GPT-4 for clinical phenotype extraction, demonstrating the comparable performance of both models in processing complex medical data. Similarly, Shi et al. investigated the representations of pre-trained models during fine-tuning, shedding light on the preservation of certain features across layers (Shi et al., 2023). These studies underline the importance of fine-tuning, which we apply to the domain of public health discourse on tobacco and e-cigarette.

Additionally, other fields have demonstrated the advantages of domain-specific fine-tuning. In the finance sector, Wilksch and Abramova created a sentiment model using a domain-specific dataset of finance-related tweets, outperforming general-purpose models in sentiment analysis (Wilksch and Abramova, 2023). Similar successes have been found in construction management (Zhong and Goodfellow, 2024) and the pharmaceutical domain, where ValizadehAslani et al. developed PharmBERT, a BERT model pre-trained on medication labels, which significantly outperformed generic models in related NLP tasks (ValizadehAslani et al., 2023). These findings highlight the potential of domain-specific fine-tuning to outperform generic models across various industries.

Recent research has also contrasted pre-trained versus fine-tuned models across different scenarios. Lossio-Ventura et al. found that fine-tuned models like OPT and ChatGPT outperformed pre-trained models when applied to health-related free-text survey data (Lossio-Ventura et al., 2024). Qin et al. introduced delta-tuning as an efficient alternative to full fine-tuning, showing comparable performance while reducing computational costs (Ding et al., 2023). These works emphasize the value of fine-tuning for domain-specific tasks, a focus that is central to our study.

While existing studies have demonstrated the potential of LLMs for sentiment analysis and public health research, a key gap remains in the comprehensive evaluation of these models for the specific domain of tobacco and e-cigarette-related social media content. Previous work has shown the effectiveness of pre-trained models like BERT for sentiment analysis in general (Elmitwalli and Mehegan, 2024; Elmitwalli et al., 2024), as well as the importance of fine-tuning for domain-specific tasks (Kocoń et al., 2023; Chung et al., 2022). However, the application of fine-tuned LLMs to the analysis of tobacco and e-cigarette discourse on social media has not been extensively explored.

Our study aims to address this gap by fine-tuning the Flan-T5 model, a lightweight yet powerful LLM, for multiple tasks in the tobacco control domain, including tobacco intent classification, e-cigarette intent classification, and sentiment analysis. This approach builds on the successes of domain-specific models seen in other fields, such as finance (Wilksch and Abramova, 2023), construction management (Zhong and Goodfellow, 2024), and the pharmaceutical industry (ValizadehAslani et al., 2023). By evaluating the performance of the fine-tuned model on both synthetic and real-world datasets, we seek to demonstrate the practical relevance and applicability of this technique for public health research and policymaking in the context of tobacco control.

## Methods

This section describes the study's overall methodology to compare fine-tuned and pre-trained Flan-T5 models for SA and intent categorization of tobacco and e-cigarette-related tweets. First the data production and preparation procedure are outlined, then the model creation and fine-tuning techniques. The evaluation metrics are then outlined, and the section concludes with the approach to data augmentation and LLM Operationalization (LLMOps) integration.

Three domain-specific datasets were created with generative AI and validated by tobacco control researchers. The initial categorization approach was piloted on a subsection of tweets, with results validated by the tobacco control researchers. Based on their feedback, iterative improvements were made to refine the generated tweets. This validation and improvement process of the prompts was repeated to develop three domain-specific classification models for analysis. Data augmentation was used to increase variety. The Flan-T5 models were fine-tuned using tobacco data to specialize for the jobs. Model performance was thoroughly assessed using accuracy, precision, recall, F1, and confusion matrices. This rigorous approach to data curation, model tweaking, and quantitative evaluation provides an appropriate framework for evaluating model capabilities on the specific twitter analytic tasks within the tobacco domain. While multilingual models offer benefits for analyzing data in different languages, the scope of this research is specifically constrained to English-language content.

### Datasets

The datasets utilized in this study were constructed to capture a diverse range of perspectives and sentiments related to tobacco and e-cigarette-related content on social media. Leveraging the advanced capabilities of GPT-4 (Sinha et al., 2024), a generative approach was employed to create three distinct datasets, each tailored for different aspects of SA and intent classification.

Hashtags in the prompts for the intent associated with tobacco and e-cigarettes were used, as hashtags act as keywords that provide context to the prompt. The hashtags were tailored to be exemplars of the types of messaging desired for the model to be able to recognize. A such, they are not hashtags identified from actual social media dialogue, but rather created for the purpose of most-effectively informing the model. By including relevant hashtags, this is essentially giving GPT-4 additional information about the topic and the intended audience. This helps the model understand the desired tone, style, and purpose of the tweet, leading to more accurate and relevant content.

The prompts for tobacco in Table 1 were designed to cover a range of perspectives. Anti-tobacco prompts highlight the negative health, financial, and/or social consequences of smoking. They may also use fear appeals and empathy to encourage quitting. The neutral prompt focuses on presenting factual information without advocating any position. Pro-tobacco prompts identify smoking as a positive, for instance as a social activity or enjoyable hobby.

**Table 1:** Prompts for the three intents of tweets associated with tobacco industry and products LLM.

**Intent**	**Prompt**
Anti-tobacco	Generate tweets associated with some strong messages about the dangers of tobacco products. Highlight the impact on health, finances, and loved ones. #KickTheHabit #LivetobaccoFree (Include statistics on health risks)
	Generate tweets that share stories of people whose lives were devastated by tobacco products. Encourage others to quit before it's too late. #alltobaccoformskills #tobaccooproductskill
	Generate tweets that capture the struggles of quitting tobacco products. Show empathy and offer support resources. #YouCanQuit #quittobacco
	Generate tweets that debunk myths about the benefits of tobacco (stress relief, etc.) and highlight the constant battle against addiction. #TobaccoHurts #BreakFreeFromAddiction
	Generate tweets that outline tobacco industry tactics to maintain and promote its business interests at the expense of health. #TobaccoTruth #DeceptiveAdvertising #CorporateGreed
Neutral	Generate tweets that present factual information about the history, cultural and health related effects of tobacco use (neither positive or negative), without promoting or advising against tobacco products.
	Generate tweets that present facts about the tobacco industry (neither positive or negative) without promoting or attacking it. #TobaccoFacts
Pro-tobacco	Generate tweets about tobacco use showing that it is a personal choice or enjoyable habit to be promoted.
	Generate tweets showing that tobacco products can be a great way to connect with people and share experiences. #SocialActivity
	Generate tweets showing that the tobacco industry can play a role in public health and should not be excluded. #HarmReductionStrategies #PublicHealthDiscussion

Similar to tobacco, the prompts for e-cigarettes as shown in Table 2 target different viewpoints. Anti-e-cigarette prompts focus on the dangers of e-cigarette, particularly for non-smokers, and may include other factors which portray e-cigarettes negatively such as criticizing the marketing of such products toward young people or raising concerns about the long-term health impacts of such products. The neutral prompt focuses on presenting factual information without advocating any position. Pro-e-cigarette prompts depict e-cigarettes as positive, for example by promoting them as a smoking cessation tool.

**Table 2:** Prompts for the three intents of tweets associated with e-cigarettes LLM.

**Intent**	**Prompt**
Anti-e-cigarette	Generate tweets that expose the dangers of e-cigarettes or vaping, especially for non-smokers. And that e-cigarettes or vaping isn't effective in tobacco cessation and can lead to dual use. #VapingKills
	Generate tweets that highlight the marketing tactics of e-cigarettes or vaping aimed at young people. #ProtectOurKids
	Generate tweets that clarify the tactics of the e-cigarettes or vaping industry and its manipulative techniques to maintain and promote its gains at the expense of health. #VapingIndustryExposed
Neutral	Generate tweets that present a balanced view of e-cigarettes or vaping, without endorsing either side. #VapingDebate #WeighTheRisks
	Generate tweets that highlight the lack of long-term research on e-cigarettes or vaping and the potential for unknown health effects, either positive or negative. #LongTermEffectsUnknown
	Generate tweets that present facts about the e-cigarettes or vaping industry without promoting or attacking it. #E-cigaretteData
Pro-e-cigarette	Generate tweets that focus on how e-cigarettes or vaping can be a tool for smokers trying to quit traditional cigarettes. And that allowing e-cigarettes or vaping is a right for free market and personal choices. #StopSmokingOptions #VapingForQuitting
	Generate tweets that share success stories of smokers who transitioned to e-cigarettes or vaping and improved their health. #QuitSmokingJourney #VapingAsHarmReduction
	Generate tweets claiming that the e-cigarettes or vaping industry can play a role in public health and should not be excluded. #PublicHealthDiscussion #HarmReductionApproach

The prompts in Table 3 focus on generating tweets with different emotional tones. Negative sentiment prompts encourage disapproval and criticism of tobacco/e-cigarettes and their health impacts. Neutral sentiment prompts aim for factual and objective statements without expressing an opinion. Positive sentiment prompts focus on the potential enjoyment or benefits associated with these products.

**Table 3:** Prompts for the three sentiments of tweets associated with tobacco/e-cigarettes LLM.

**Intent**	**Prompt**
Negative	Generate tweets with negative sentiment about tobacco or vaping or e-cigarette products and industry.
Neutral	Generate tweets with neutral sentiment about tobacco or vaping or e-cigarette products and industry.
Positive	Generate tweets with positive sentiment about tobacco or vaping, or e-cigarette products and industry.

The GPT-4 hyperparameters control various aspects of the language model's text generation behavior (Katz et al., 2024). Careful selection of these values is important to achieve high-quality tweet generation for the tobacco and e-cigarette-related datasets as shown in Table 4.

- Temperature: The temperature of 0.7 was chosen to maintain a good balance between creativity and coherence in the generated tweets. This value allows for some diversity while ensuring the tweets remain relevant and focused on the given prompts.
- Top-*p* sampling: A top-*p*-value of 0.8 was selected to generate diverse and creative tweets while maintaining coherence and relevance to the prompts.
- Frequency penalty: A frequency penalty of 0.4 helps discourage repeating common words or phrases too frequently across tweets. This maintains diversity and reduces redundancy in the generated content.
- Presence penalty: Setting the presence penalty to 0.3 discourages reusing words or phrases already generated, even once. This encourages a wider range of vocabulary and concepts across tweets, promoting more diverse output.

**Table 4:** The hyperparameters of the GPT-4 LLM used for tweets generation.

**Hyperparameter**	**Explanation**	**Chosen value**
Temperature	Maintains balance between creativity and coherence	0.7
Top-p sampling	Generates diverse and creative tweets while staying relevant	0.8
Frequency penalty	Discourages repetitive words/phrases	0.4
Presence penalty	Encourages exploration of vocabulary and concepts	0.3

**Figure 1:**
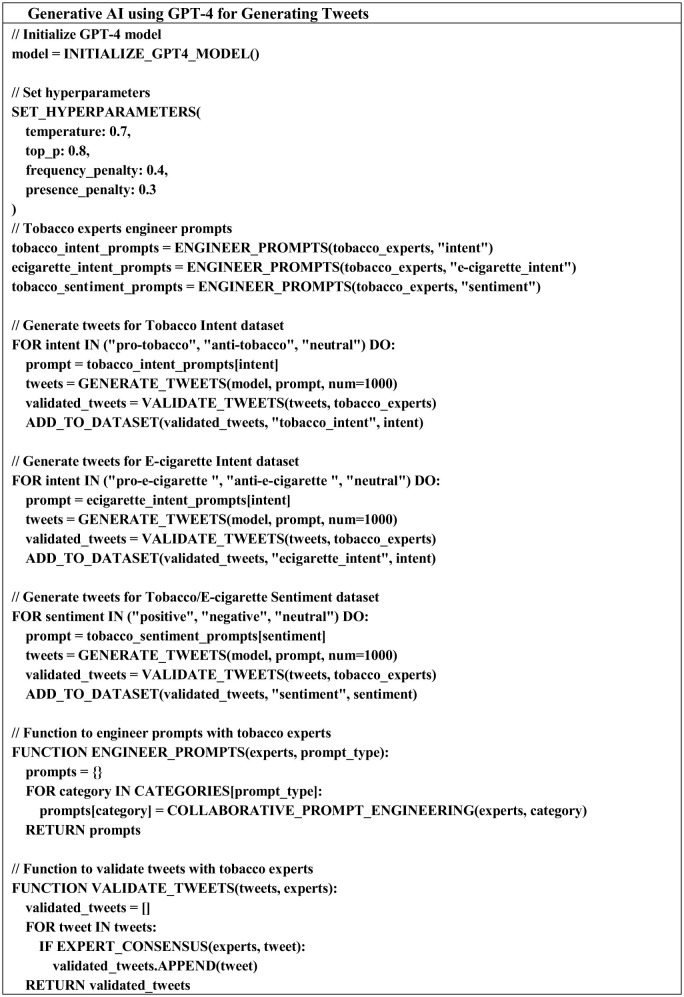
The tweets generation pseudo code using Generative AI.

The pseudo code shown in Figure 1 illustrates how GPT-4 was utilized to generate a large dataset of tobacco-related tweets. It creates tweets with specific sentiments (positive, negative, neutral) and intents (pro-tobacco, anti-tobacco, and neutral) using tailored prompts. Validation by tobacco control researchers ensure the generated tweets are relevant and realistic, ultimately building a high-quality dataset for SA of tobacco discussions on social media.

#### Tobacco intent LLM dataset

This dataset explores purpose/advocacy in tobacco tweets. Tweets were categorized as anti-tobacco, neutral, or pro-tobacco based on objective tone. GPT-4 generated 3,000 tweets (1,000 per category) capturing a wide range of intents. A random subsample was validated by tobacco control specialists, with iterative improvements to prompts/generation guided by feedback. This facilitates investigating tobacco perspectives on social media.

#### E-cigarette intent LLM dataset

This examines intent in e-cigarette tweets. Tweets are classified as anti-e-cigarette, neutral, or pro-e-cigarette based on underlying advocacy. GPT-4 generated 3,000 tweets (1,000 per category) representing diverse viewpoints. As with Tobacco Intent, a random sample underwent domain validation to refine generation. This supplements tobacco analysis via comparing tobacco and e-cigarettes product discussions.

#### Tobacco/e-cigarette sentiment LLM dataset

This investigates sentiment in tobacco and e-cigarette tweets. Tweets were carefully sorted into positive, negative, or neutral emotional categories by assessing tone. GPT-4 generated 3,000 sentiment-labeled tweets (1,000 each) to fully capture opinions. A random sample underwent domain review to ensure sentiment categories were applied accurately based on emotional tone. This methodology and scale facilitate robust investigation of social media perspectives on these topics. This method guarantees a full depiction of opinions regarding tobacco and e-cigarettes.

### Dataset validation

To ensure the authenticity and relevance of the generated tweets, two the of authors with tobacco control research expertize designed prompts aimed at authentically capturing tobacco and e-cigarette-related discussions on social media. To validate the quality and relevance of the generated tweets, the researchers utilized a standardized rubric to evaluate a random sample from the dataset. The rubric assessed tweets based on criteria such as relevance to tobacco topics, authentic language and tone, factual accuracy, and accurate portrayal of sentiment or intent. Specialists applied a 5-point Likert scale for each criterion, requiring a score of 4 or above for inclusion. When initial evaluations differed, a third researcher analyzed the tweet and facilitated consensus through discussion. This process revealed opportunities to refine around 15% of tweets, either through small edits or generating replacement content. Specialists also provided iterative feedback on prompts, incrementally improving the GPT-4 output quality.

While full validation of all 9,000 tweets was infeasible at this scale, this sample review process established confidence in the overall quality of the dataset. The involvement of domain specialists at each stage ensured that the generated content accurately represented real-world tobacco and e-cigarette-related discussions on social media. This validation methodology not only verified the quality of individual tweets but also contributed to the overall dataset development process, demonstrating that techniques like GPT-4 can produce valuable data when informed by domain expertize. The resulting validated datasets facilitate a comprehensive exploration of fine-tuned Flan-T5 models' ability to analyze sentiment and classify intent in tobacco and e-cigarette-related tweets.

In addition to the synthetic datasets, we incorporated 1,155 real-world tweets collected around the duration of the Ninth Conference of the Parties to the WHO Framework Convention on Tobacco Control (COP9; Elmitwalli et al., 2024). These tweets were filtered to include only English-language content and underwent standard pre-processing, which involved the removal of URLs, user mentions, and unnecessary formatting elements. GPT-4 was employed to analyze and assist in selecting relevant tweets for each LLM by classifying them based on tobacco intent, e-cigarette intent, and sentiment. Approximately 347 tweets were annotated for the sentiment LLM, 207 for the tobacco intent LLM, and 131 for the e-cigarette intent LLM by two domain specialists, following the same rigorous evaluation process applied to the synthetic dataset. Among those tweets, only seven tweets were associated with anti-e-cigarette intent and nine tweets were associated with pro-tobacco intent. This real-world data was then used to test the three fine-tuned LLMs, providing additional validation for generalizability and model robustness.

### Data augmentation

After the datasets were constructed, we used data augmentation techniques to increase the diversity and amount of our training data. As a result, three augmentation approaches were applied to increase variety and extend the data. First, the synonym substitution strategy entailed replacing certain terms in the original tweets with their synonyms. This approach can create linguistic differences while maintaining the overall message of the tweets (Kapusta et al., 2024). Second, the back translation approach included translating first, then back-translating. The tweets are translated into Indonesian and then back into the original language. Indonesian was selected due to its distinct grammatical structure from English and widespread translation support, which helps generate more diverse yet semantically consistent variations. This approach attempts to produce fresh copies of tweets with various language and expressions (Chen et al., 2024). Finally, the contextual word replacement (CWR) approach uses a pre-trained BERT model to replace words in tweets with contextually relevant replacements. This method intends to generate more diverse and contextually appropriate tweet versions, with the ability to capture many elements of mood and language use (Bencke and Moreira, 2024). When combined with the original datasets, the use of these three augmentation approaches is expected to double their size. This augmentation technique is used to increase the variety of the dataset, resulting in a more thorough representation for future analysis and model training.

The data augmentation procedure enlarged the original datasets. Starting with 3,000 tweets per model (1,000 per category), the augmentation strategies tripled the size of each dataset, yielding over 12,000 tweets per model. To achieve rigorous training and evaluation, 90% of the data was allocated for training and validation, with 10% reserved for testing. Within the 90%, 80% was used for training and 10% for validation. This resulted in a training set of ~9,600 tweets, a validation set of 1,200 tweets, and a test set of 1,200 tweets per model. During fine-tuning, the model was trained for 10 epochs, with checkpoints saved at the end of each epoch. The best-performing model, based on validation performance, was selected for final evaluation on the test set. This approach ensured a robust model selection process.

The assessed augmented tweets were integrated into the training pipeline to fine-tune the Flan-T5 SA and classification models. The inclusion of tobacco control researchers in the review process helped to refine and enhance the algorithms' ability in capturing the intricacies of sentiment in tobacco and e-cigarette-related tweets. Furthermore, using domain specialists in the assessment process guarantees that the models are trained on high-quality data and can evaluate the sentiment and categorization of tobacco and e-cigarette-related tweets in a thorough manner.

### Performance enhancement approaches

Different techniques are employed with LLMs to improve adaptation and task-specific performance. These include zero-shot learning, few-shot learning, fine-tuning, and instruction/prefix tuning. Zero-shot learning refers to a model's capacity to generalize to new classes or categories that it did not encounter during training. Unlike classical supervised learning, which trains a model on labeled examples from all predicted classes, zero-shot learning trains the model on a different set of classes than those seen during testing. The model learns to comprehend the link between visible and unseen classes, allowing it to correctly classify unseen samples (Hou et al., 2024). Few-shot learning allows LLMs to learn fast from a small number of examples, letting them to generalize to comparable tasks with minimal additional training. This method is especially effective when labeled data for a given job is limited (Wang et al., 2024). LLMs are fine-tuned by training them on task-specific data to increase their performance on selected tasks. This method enables more complete adaptation to domain-specific requirements, although it can be computationally demanding for larger models (Lin et al., 2024). Instruction or prefix tuning directs LLMs during inference by giving explicit instructions or prompts that allow control over the resulting output. This method is particularly useful for controlling the model's behavior without considerable retraining (Zou et al., 2024). These techniques cater to a variety of circumstances, providing flexibility and efficiency in the use of LLMs for a wide range of tasks with varying amounts of data availability and output requirements.

In this study, fine-tuning is used as the major technique to improve the performance of the Flan-T5 model for the specific objectives of SA and intent categorization of tobacco and e-cigarette-related tweets. While other techniques, such as zero-shot learning, few-shot learning, and instruction tuning, have their merits, fine-tuning was selected because it allows adjustment of the model more thoroughly to the domain-specific data. This option allows the use of Flan-T5's pre-trained knowledge while adapting it to the unique subtleties of tobacco and e-cigarette-related emotion and intent categorization.

### Flan-T5

T5, also known as the Text-to-Text Transfer Transformer, is a sophisticated design in the field of natural language processing. Unlike traditional models, which are task-specific, T5 employs a text-to-text approach, defining each task as input-output transformations. This means that, independent of the job at hand, such as translation, question answering, or classification, the model is trained to produce the required output text from the input text. This unified architecture enables the use of a single model, loss function, hyperparameters, and other components across a wide range of tasks. It offers a more streamlined and efficient approach to natural language processing by reducing the requirement for task-specific models and allowing for faster model deployment and transferability (Vaishnavi et al., 2024).

T5 contains significant enhancements over its predecessor, BERT. It is meant to be computationally efficient, making it faster and less resource-intensive to train and deploy than models such as GPT-3. Furthermore, T5 is pre-trained on a variety of unsupervised and supervised activities, with each task converted to a text-to-text format. This allows the model to learn from a wide variety of inputs and adapt to different tasks without requiring task-specific modifications. It has significant skills in zero-shot learning, few-shot learning, and chain of thought reasoning, making it ideal for a wide range of natural language applications.

**Figure 2:**
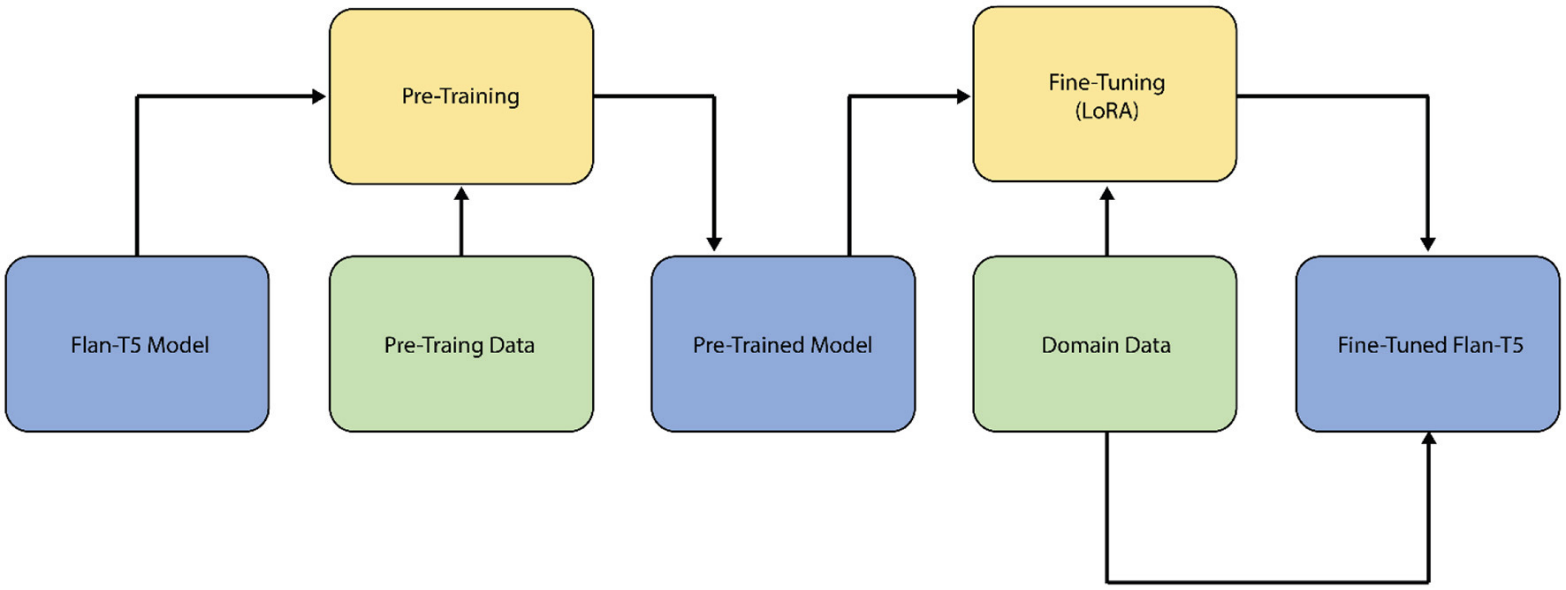
The Flan-T5 LLM fine-tuning.

The Flan-T5 model, on the other hand, is a pre-trained version of T5 that is trained on a broad range of tasks, both supervised and unsupervised, with each task translated into a text-to-text format, as illustrated in Figure 2. During the training phase, Flan-T5 is exposed to a large amount of text data and taught to identify missing words in input texts using a fill-in-the-blank method. This training procedure is repeated several times until the model can create text that roughly mimics the input data.

Furthermore, Flan-T5 is a flexible model that excels in text classification tasks, allowing for the classification of text into categories such as spam or non-spam, positive or negative sentiment, or particular themes such as politics, sports, and entertainment. This functionality is useful for a variety of applications, including content filtering, customer support, and personalized suggestions. While Flan-T5 has additional features such as text creation, text summarization, question-answering, translation, and conversational AI, the fine-tuning for this study is concentrated on the text categorization component.

### Low-rank adaptation

We employed Low-Rank Adaptation (LoRA), a fine-tuning technique that reduces the number of trainable parameters by decomposing weight updates into lower-rank matrices. This approach significantly minimizes the parameter space compared to full fine-tuning, inherently limiting the model's capacity to overfit to the training data. Importantly, LoRA does not modify the original pre-trained weights; instead, it adds small, trainable low-rank matrices to each layer, which helps preserve the general knowledge learned during pre-training, acting as a form of regularization (Pathak et al., 2023). We chose LoRA for its ability to enhance computational efficiency while enabling effective domain adaptation in our LLMs. This allowed us to fine-tune the Flan-T5 model on domain-specific datasets (tobacco and e-cigarette tweets), ensuring improved performance without excessive computational costs. Furthermore, LoRA facilitates adaptability to new tasks or domains without drastically altering the model's overall structure, thus maintaining the good generalization properties of the original model.

Therefore, LoRA method is leveraged in this work to efficiently fine-tune the large pre-trained Flan-T5 model on the desired downstream tasks. LoRA operates by freezing the weights of the original Flan-T5 model and introducing small trainable low-rank matrices into the query, key, value, and output projection layers (Hu et al., 2023). These low-rank matrices introduce much fewer trainable parameters (~4.72 million) than the original Flan-T5 model, which had 250 million total parameters. During training, only the low-rank matrices are updated, while the initial Flan-T5 weights remain unchanged. This technique allows for very efficient fine-tuning on fresh datasets by drastically lowering the number of trainable parameters, all while retaining the original model's expressive capacity and performance. Following training, these low-rank adaptations are merged back into their corresponding layers in the Flan-T5 model, resulting in no computational burden at inference time. Thus, the altered Flan-T5 model preserves its original capabilities while being lightly modified for the present specialized purposes. This approach successfully adapts big pre-trained models, such as Flan-T5, to downstream applications without the need for substantial retraining. Furthermore, using LoRA for fine-tuning is extremely helpful in adjusting the huge pre-trained Flan-T5 model to the desired unique needs. This strategy can enable efficient fine-tuning without the risk of catastrophic forgetting or overfitting, allowing the model to maintain broad language knowledge while accumulating domain-specific expertize.

### Classification evaluation

Classification evaluation metrics are critical tools for determining the performance and efficacy of classification algorithms. These measures give essential information about the model's accuracy, precision, recall, and general predictive skills. The accuracy of SA and text categorization was determined by comparing model predictions to ground truth labels in the dataset. Both models were assessed based on their ability to accurately categorize tweets into sentiment categories (negative, neutral, and positive) as well as larger classifications (anti-tobacco, neutral, and pro-tobacco) and (anti- e-cigarette, neutral, and pro- e-cigarette). Precision, which evaluates the proportion of accurately categorized occurrences among anticipated positive instances, was also tested to verify the models' dependability in recognizing certain moods or perspectives. The recall metric was used in this study to evaluate the models' ability to accurately identify all instances of a specific sentiment or class. It evaluated the models' capacity to catch and properly categorize the full range of sentiments expressed in tobacco-related tweets. The F1-score, which takes into consideration both precision and recall, gave an overall assessment of the models' performance by accounting for both the accuracy and completeness of their predictions (Naidu et al., 2023).

### LLMOps automation and orchestration

**Figure 3:**
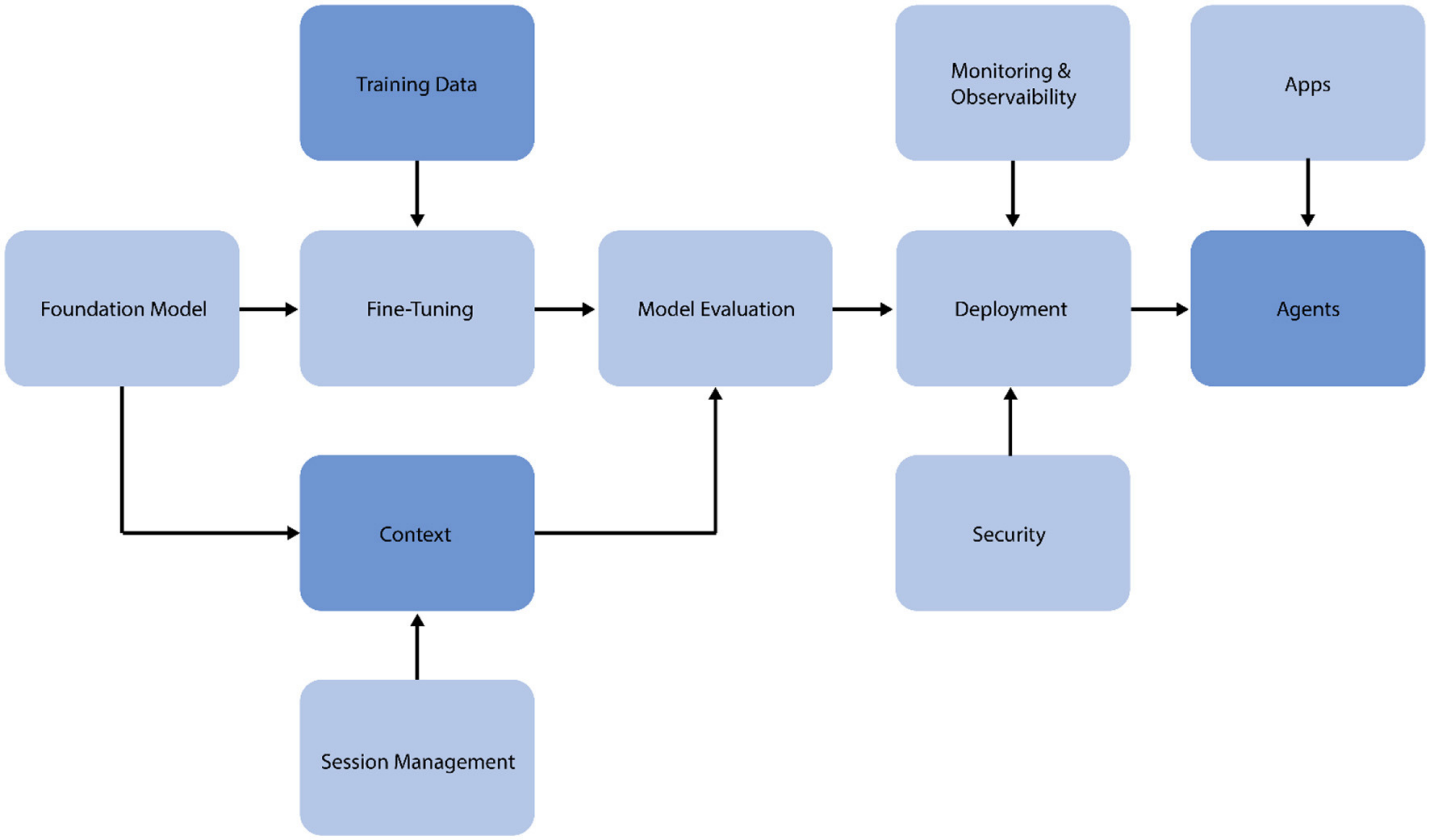
The LLMOps pipeline for the proposed system.

This section outlines the implementation of a Large Language Model Operations (LLMOps) pipeline for fine-tuning and deploying the Flan-T5 model for SA and intent classification of tobacco/e-cigarettes-related tweets (Chen, 2024). The LLMOps approach ensures scalability, reproducibility, and efficient management of the model lifecycle, as shown in Figure 3.

The pipeline begins with data preparation, where the generated datasets, including the original tweets and augmented versions, are processed, and formatted for model input. This stage involves tokenization, encoding, and splitting the data into training and validation sets. Model fine-tuning is conducted using the LoRA method. This approach entails freezing the initial model weights, including trainable low-rank matrices into specified layers, and optimizing hyperparameters such as learning rate, batch size, and epoch count. Training progress is tracked using parameters such as accuracy, precision, recall, and F1-score. After fine-tuning, the model is evaluated on a hold-out test set to determine its performance in SA and intent classification tasks. Confusion matrices are constructed to offer extensive information about the model's categorization skills.

After successful fine-tuning and validation, the model is deployed to the Hugging Face platform. This deployment technique enables other researchers to easily access and use Hugging Face's Python API, version control, model tracking, and scalable serving of model predictions. The deployed model is constantly evaluated for performance in real-world circumstances. Feedback loops are constructed to gather or produce fresh data and retrain the model as needed, ensuring that it remains relevant and accurate over time. The feedback loops are closely integrated with context and session management systems, allowing the model to adapt dynamically to new data and evolving research needs.

The LLMOps pipeline uses automation technologies to simplify these procedures, decreasing manual intervention and possible mistakes. This technique enables quick experimentation, allowing for efficient testing of various model configurations and fine-tuning procedures. By using this LLMOps pipeline, we ensure that our fine-tuned Flan-T5 model for tobacco and e-cigarette-related tweet analysis is not only high-performing, but also maintainable, scalable, and adaptive to changing research demands in public health and tobacco control.

## Results and discussion

The selection and adjusting of hyperparameters were critical in optimizing the present fine-tuned Flan-T5 model, as shown in Table 5. A methodical approach to hyperparameter modification was taken, combining grid search and manual fine-tuning. The LoRA rank (r) and alpha were adjusted to 64 based on empirical investigations indicating that these values provide a fair mix of model flexibility and computing efficiency for models of size. A lower learning rate (3e-4) was used to maintain consistent training, which is especially crucial when doing LoRA on a previously trained model. The batch size of 32 was chosen as the maximum number that could be stored in GPU memory while ensuring training stability. Initially a high number of epochs of 10 was selected, but early stopping with a three-epoch patience was implemented to prevent overfitting. This was especially important given the model's strong performance, which prompted worries about overfitting. The AdamW optimizer was chosen because of its ability to handle sparse gradients in NLP workloads. The evaluation frequency was reduced to once per epoch to create a balance between extensive monitoring and computational efficiency. The validation F1 score was chosen as the primary model selection statistic since it balances accuracy and recall, with ablation tests used to assess the impact of different hyperparameters, focusing on LoRA-specific aspects (rank, alpha, and dropout). This method enabled efficient fine-tuning of the model while learning about its sensitivity to various hyperparameter values.

**Table 5:** The Flan-T5 fine-tuning hyperparameters.

**Hyperparameter**	**Description**	**Value**
Model architecture	The specific model being fine-tuned with Lora	FLAN-T5-base
LoRa rank (r)	Dimensionality of LoRa decomposition	64
LoRa alpha (lora_alpha)	Scaling factor for LoRa update	64
LoRa dropout (lora_dropout)	Dropout rate applied to LoRa update	0.05
Learning rate	The learning rate used for optimization	3e-4
Batch size	The number of samples per batch	32
Number of training epochs	The total number of passes over the training data	10
Optimizer	The algorithm used for updating model weights	AdamW
Evaluation frequency	How often model performance is evaluated on held-out data	Every epoch
Early stopping criteria	The patience and metric used for early stopping	Stop after 3 epochs without improvement on validation F1
Monitoring metric	The metric tracked and used to select the final model	Validation F1
Hardware/software environment	The computation setup used for experiments	RTX A4000/Python

**Figure 4:**
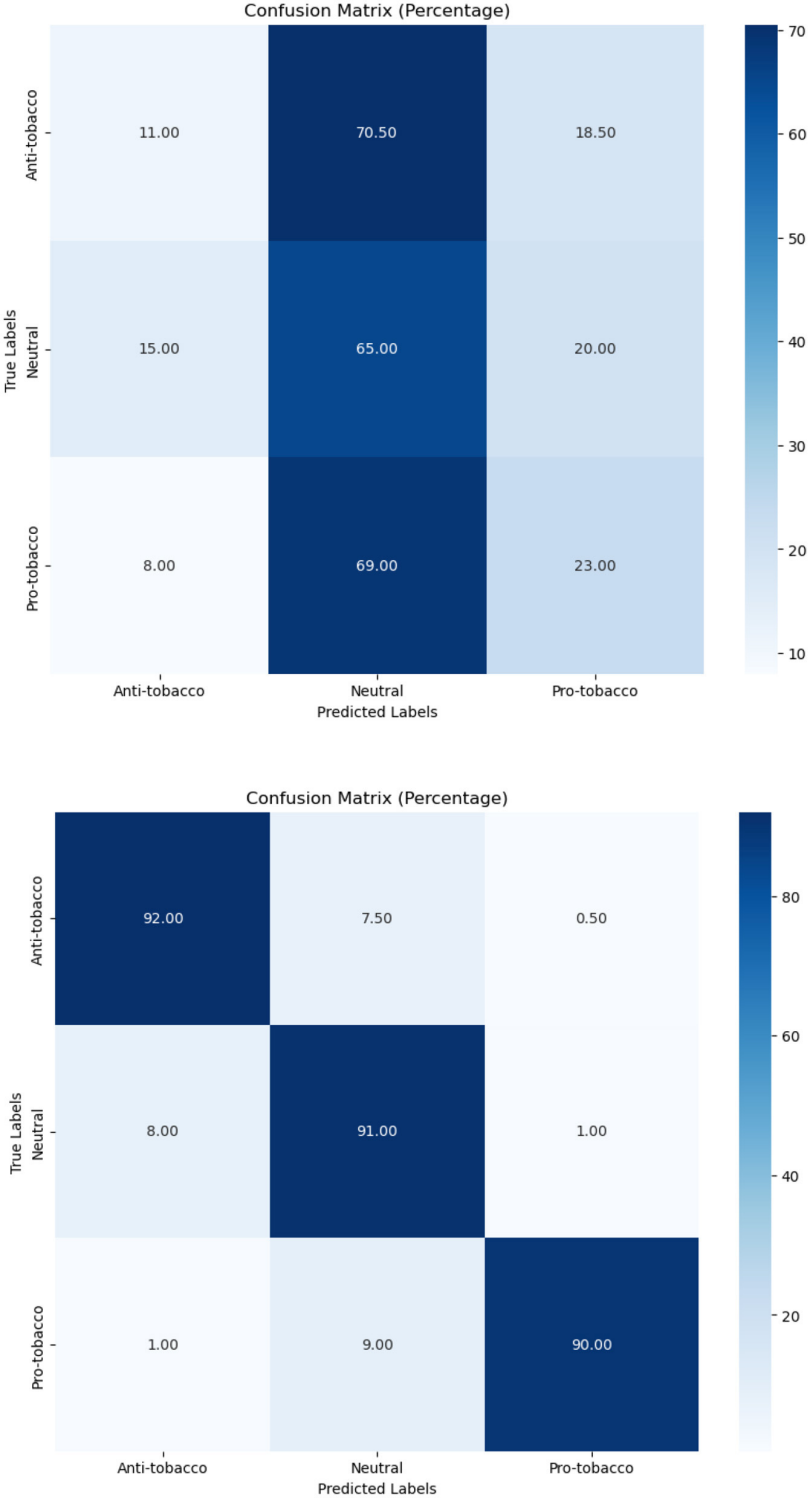
Pre-trained and fine-tuned Flan-T5 performance for tobacco intent classification.

**Figure 5:**
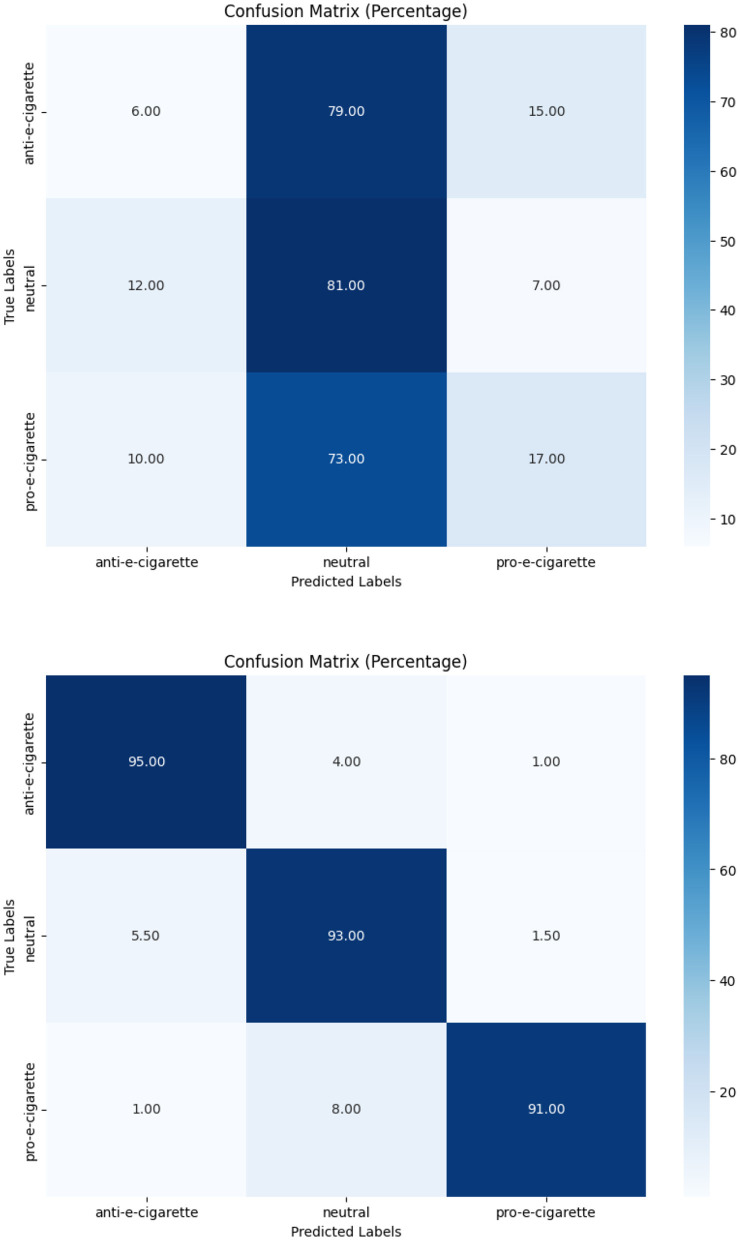
Pre-trained and fine-tuned Flan-T5 performance for e-cigarette intent classification.

**Figure 6:**
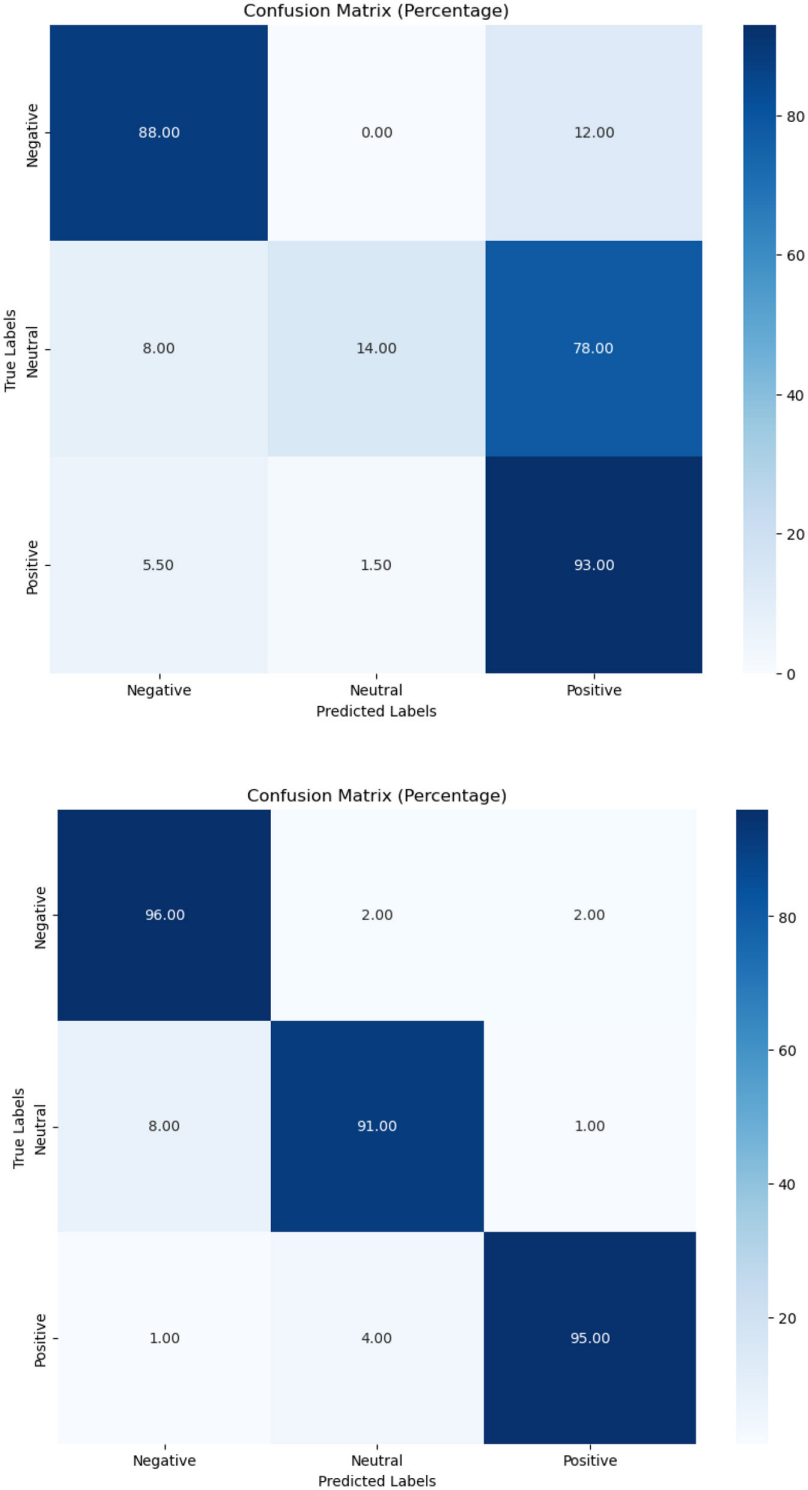
Pre-trained and fine-tuned Flan-T5 performance for tobacco/e-cigarette SA.

In terms of tobacco intent categorization, the fine-tuned model performed well, with F1-scores of 0.92, 0.88, and 0.94 for anti-tobacco, neutral, and pro-tobacco categories, respectively, as shown in Table 6. This result is a significant increase over the pre-trained model's accuracy of 0.33. The confusion matrices shown in Figures 4–6 give a visual picture of this improvement, showing classification with high accuracy for the fine-tuned model in sharp contrast to the pre-trained model's inability to differentiate between categories.

**Table 6:** The pretrained and fine-tuned LLMs performance.

	**Tobacco intent**			**E-cigarette intent**			**Tobacco/e-cigarette sentiment**		
	**Anti-tobacco**	**Neutral**	**Pro-tobacco**	**Anti-e-cigarette**	**Neutral**	**Pro-e-cigarette**	**Negative**	**Neutral**	**Positive**
F1-score^a^	0.92	0.88	0.94	0.94	0.91	0.94	0.94	0.92	0.96
Precision^a^	0.91	0.85	0.98	0.94	0.89	0.97	0.91	0.94	0.97
Recall^a^	0.92	0.91	0.90	0.95	0.93	0.91	0.96	0.91	0.95
Accuracy^a^	0.91			0.93			0.94		
Flan-T5 accuracy^b^	0.33			0.36			0.65		

^a^For the fine-tuned Flan-T5 LLMs.

^b^For the pre-trained Flan-T5 LLMs.

In e-cigarette intent classification, the fine-tuned model performed similarly to the tobacco intent classifier, with F1-scores of 0.94, 0.91, and 0.94 for anti- e-cigarette, neutral, and pro- e-cigarette, respectively (see Table 6). This represents a significant increase over the pre-trained model's accuracy of 0.36. The confusion matrices in Figures 4–6 clearly highlight this improvement, demonstrating the fine-tuned model's capacity to produce extremely accurate classification performance across all categories.

The SA job for tweets on tobacco and e-cigarette validated the fine-tuned model's performance. It attained F1-scores of 0.94, 0.92, and 0.96 for negative, neutral, and positive attitudes, respectively (Table 6), well above the pre-trained model's accuracy of 0.65. The confusion matrices in Figures 4–6 give visual proof of this improvement, demonstrating the fine-tuned model's ability to differentiate between sentiments with superior precision. Several fundamental aspects contribute to the significant performance increases reported upon fine-tuning. First, domain-specific training enabled the model to learn specialized language, contextual subtleties, and semantic complexities relevant to tobacco and e-cigarette-related social media speech. This focused learning allowed the model to catch nuanced language signals and contextual information, which are critical for effective categorization in this specialized subject.

Second, fine-tuning enabled task-specific optimization, allowing the model's pre-trained information to be adapted to the particular problems of intent classification and SA in the tobacco context. This targeted strategy enabled the model to use its wide language knowledge while improving its talents for these specific tasks. The use of a thoroughly chosen and balanced dataset for fine-tuning is believed to have contributed significantly to the model's ability to perform consistently well in all categories. This balanced method guaranteed that the model was exposed to a representative variety of samples from each category, preventing bias toward any one class.

These findings highlight the important role of domain-specific fine-tuning in obtaining high performance in specialized NLP tasks. The fine-tuned Flan-T5 model exhibits an impressive capacity to catch the intricacies of tobacco-related social media discussions, which is critical for accurate SA and intent categorization in this field. While fine-tuned model's strong performance may signal a risk of overfitting to the training data, the use of a separate validation set mitigated this risk. By splitting 90% of the data into 80% for training and 10% for validation, we ensured that the model was evaluated on unseen data during the training process. This approach, combined with selecting the best-performing model based on validation performance, helped improve generalizability and reduce overfitting. While the evaluation results show strong generalizability within the experimental dataset using the remaining 10% for testing, the model was only exposed to a limited sample of social media discussions. To more rigorously assess this risk, we have tested on a held-out evaluation set comprising real-world tobacco/e-cigarette tweets. As broader, more diverse test collection would provide a robustness check and further validate the model's ability to generalize beyond the specific data it was fine-tuned on. Such analyses are important to establish reliability and identify opportunities for increased robustness as domain models are applied in real-world settings.

The significant performance gap between the pre-trained and fine-tuned models also raises interesting questions about the nature of transfer learning in Large Language Models. While the pre-trained model's poor performance on these specific tasks might seem surprising given its broad language understanding, it highlights the importance of domain-specific knowledge in specialized classification tasks. This observation opens avenues for future research into more efficient methods of adapting LLMs to new domains while retaining their general capabilities.

While the validation process ensured high quality for the generated dataset, it is important to acknowledge that synthetic data may not capture all the nuances of real-world social media content, such as slang, cultural context, or rapidly changing trends. Therefore, we have tested the models on real, annotated Twitter data from the COP9 event. The evaluation of the fine-tuned LLMs on the COP9 dataset of real annotated tweets reveals strong performance across sentiment and intent classification tasks, providing crucial validation of the models' generalizability beyond synthetic data.

**Figure 7:**
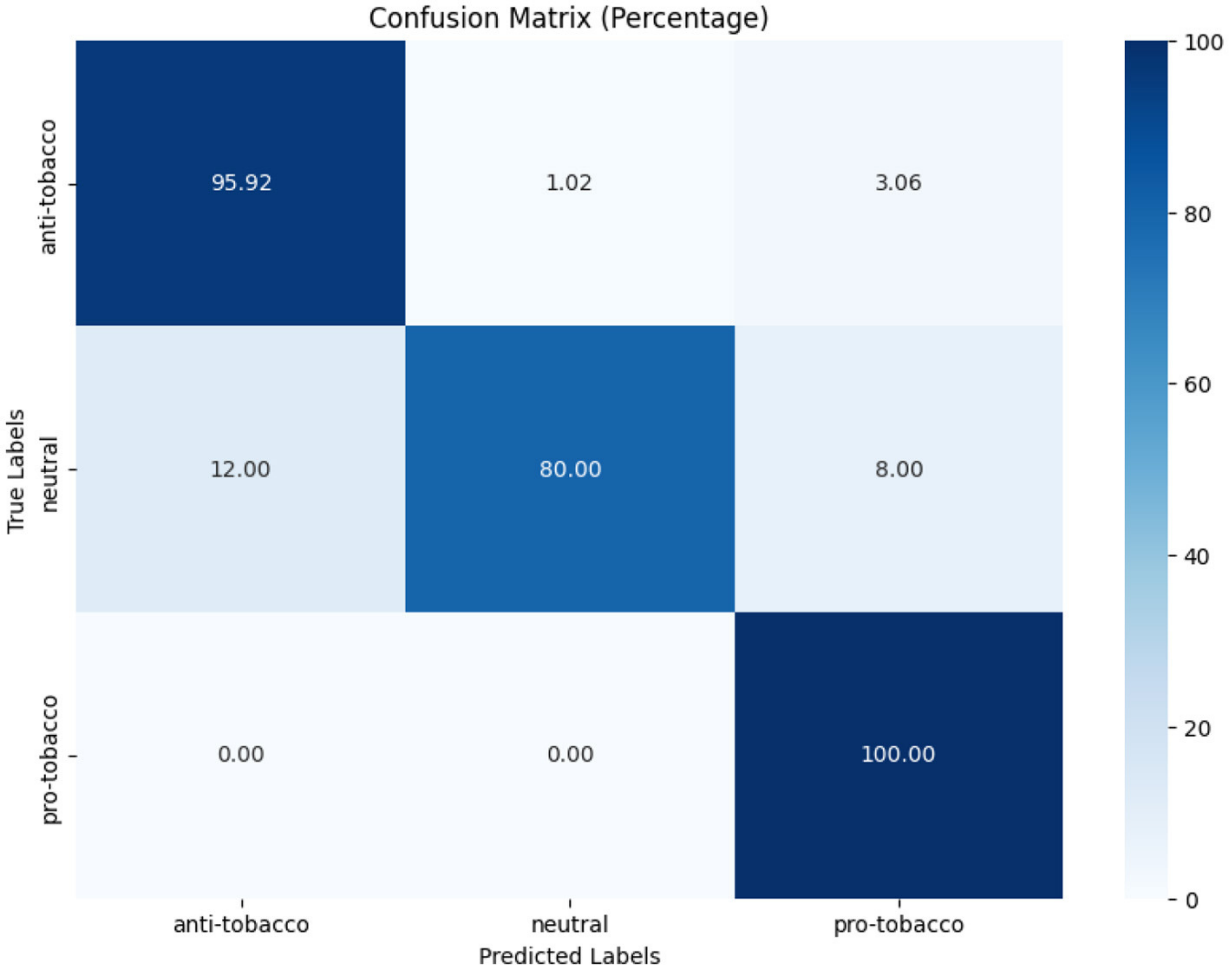
Tobacco intent model performance on real annotated tweets.

**Figure 8:**
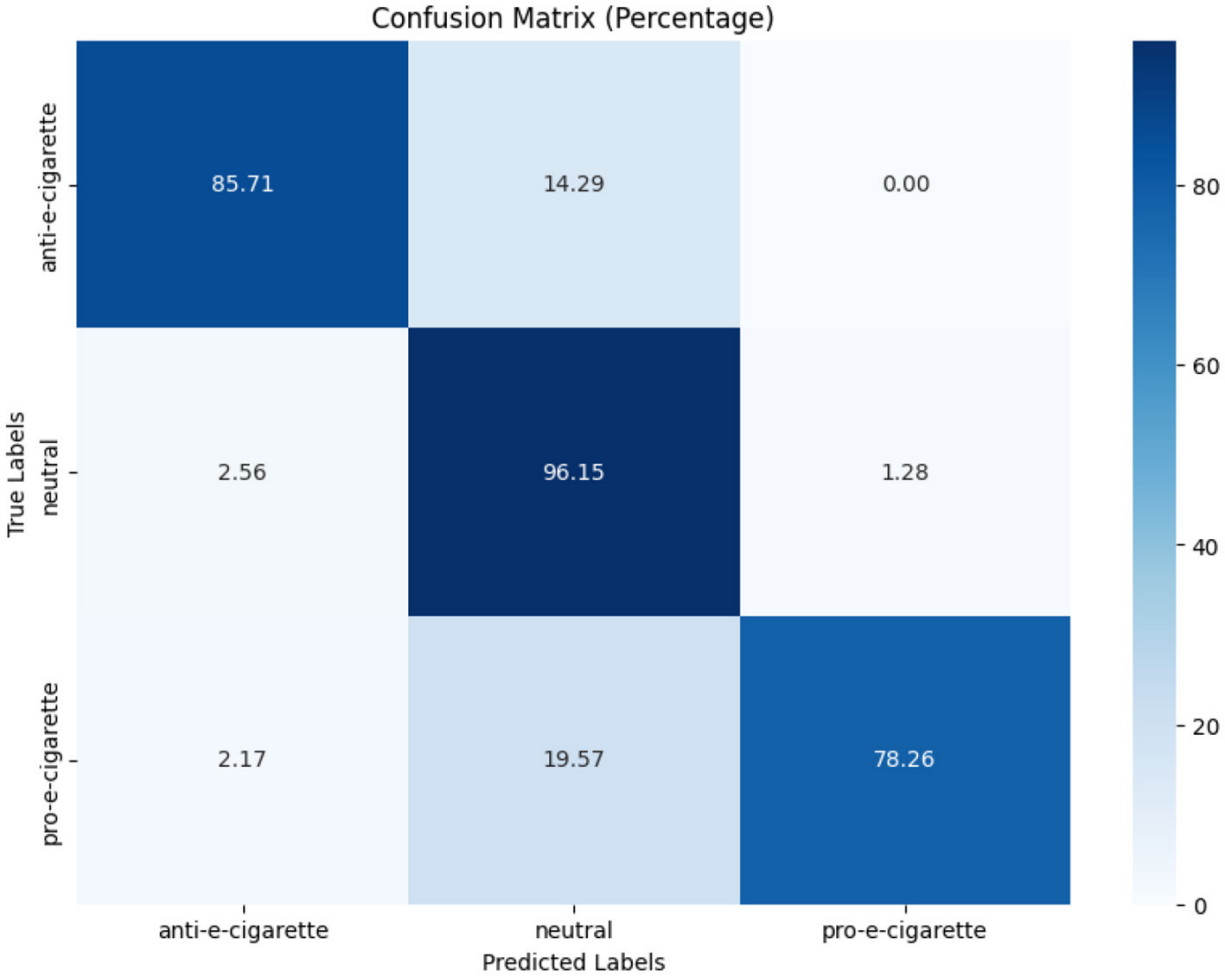
E-cigarette intent model performance on real annotated tweets.

**Figure 9:**
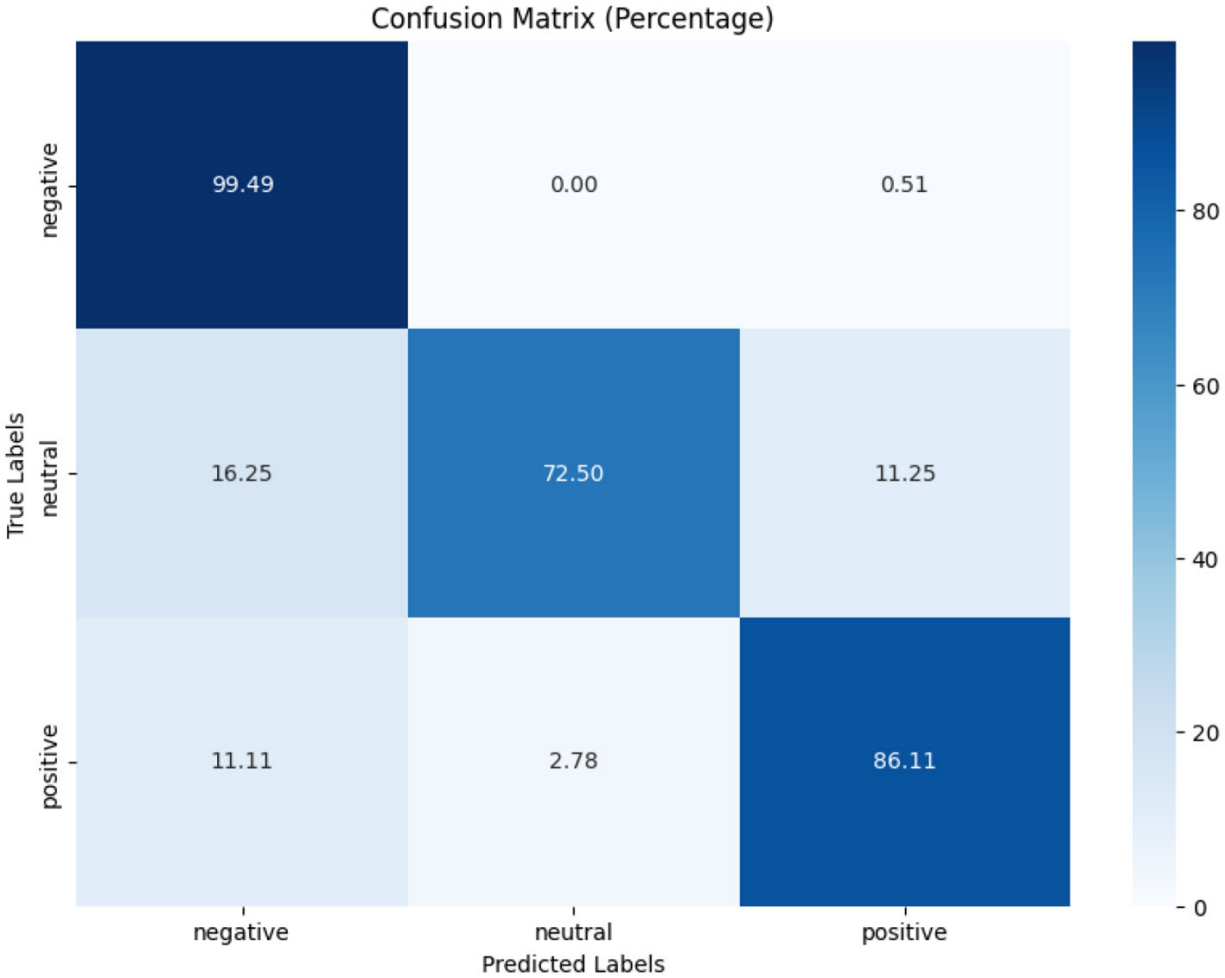
Sentiment model performance on real annotated tweets.

Figures 7–9 depict the confusion matrices for the fine-tuned models on real annotated tweets from the COP9 dataset, offering a detailed visual summary of their classification performance. Figure 7 focuses on tobacco intent classification, where the model achieved high accuracy in classifying anti-tobacco and pro-tobacco tweets, while the neutral category had a lower percentage of accurate classifications. Figure 8 presents the confusion matrix for e-cigarette intent classification, showing strong performance in classifying neutral e-cigarette intent tweets followed by anti-e-cigarette then pro-e-cigarette intent. Figure 9 illustrates the sentiment classification results, where the model performed particularly well in identifying negative sentiments, followed by positive then neutral sentiments. These confusion matrices reveal the fine-tuned models' overall robustness.

While Table 7 show the fine-tuned models that demonstrated good F1-scores, particularly in tobacco intent classification, with scores of 0.89 for anti-tobacco, 0.96 for neutral, and 0.92 for pro-tobacco tweets. These results indicate that the model effectively captures domain-specific language and intent variations. While the results for e-cigarette intent classification were more varied, with F1-scores of 0.67 for anti-e-cigarette, 0.86 for neutral, and 0.75 for pro-e-cigarette tweets, the lower performance in anti-e-cigarette classification suggests that it faces challenges, possibly due to subtler nuances or the relative rarity of anti-e-cigarette in the dataset. For sentiment analysis, the fine-tuned model performed consistently well, achieving F1-scores of 0.90 for negative, 0.99 for neutral, and 0.95 for positive sentiments. The high precision and recall scores reflect the model's robustness in capturing diverse emotional tones within the real tweets, further underscoring its utility in sentiment analysis.

**Table 7:** Evaluation of fine-tuned LLMs on real annotated tweets (COP9 dataset).

	**Tobacco intent**			**E-cigarette intent**			**Tobacco/e-cigarette sentiment**		
	**Anti-tobacco**	**Neutral**	**Pro-tobacco**	**Anti-e-cigarette**	**Neutral**	**Pro-e-cigarette**	**Negative**	**Neutral**	**Positive**
F1-score	0.89	0.96	0.92	0.67	0.86	0.75	0.90	0.99	0.95
Precision	0.98	0.80	0.88	0.88	0.96	0.92	0.97	0.73	0.83
Recall	0.45	1.00	0.62	0.97	0.78	0.87	0.86	0.85	0.86
Accuracy	0.88			0.89			0.91		

Overall, these results confirm that the fine-tuned LLMs, validated on real-world data, maintain strong performance in both intent and sentiment classification. This strengthens the models' applicability to practical public health research, where accurate analysis of social media content can inform policy-making and targeted interventions.

## Conclusion

This work demonstrates the potential of fine-tuned Flan-T5 models for evaluating tobacco and e-cigarette-related social media information, making significant contributions to both research methodology and practical applications in public health and tobacco control. The improvements in accuracy, precision, and recall across tasks (tobacco intent classification, e-cigarette intent classification, and SA) demonstrate the efficacy of domain-specific fine-tuning in enhancing the capabilities of Large Language Models. The fine-tuned algorithms' ability to categorize tweets and attitudes provides deeper insights into complex tobacco debates on social media, offering a level of understanding that was previously difficult to achieve at scale. The effective use of LoRA in fine-tuning the Flan-T5 model also demonstrates a method for adapting pre-trained Large Language Models to specific domains and tasks while maintaining computational efficiency. This is especially crucial for resource-limited public health and tobacco control contexts, where high performance is needed despite limited resources.

The effectiveness of this strategy suggests its broader applicability in other specialized disciplines requiring domain-specific language analysis. These findings have important implications for tobacco research and policy, offering researchers and policymakers a powerful tool to monitor public discourse in real time. This enhanced analytical capacity can improve the evaluation of tobacco control initiatives, enabling more responsive and data-driven policy decisions.

While real-world data presented challenges due to the lower frequency of rarer intents, such as pro-tobacco and anti-e-cigarette, the use of synthetic datasets for training effectively balanced these classes. By addressing the rarity of specific categories, synthetic data enhanced the model's ability to generalize, demonstrating the value of data augmentation in overcoming real-world imbalances. This approach ensured robust performance even when encountering rare intents in social media discussions. Additionally, the accurate classification of pro-tobacco and anti-e-cigarette content opens new possibilities for developing targeted public health initiatives. Understanding the nuances of how tobacco and e-cigarettes are discussed on social media enables health communicators to create more resonant messages that combat misinformation and promote healthy behaviors.

Nonetheless, the fine-tuned LLMs were further validated through real-world data from the COP9 dataset, confirming their ability to accurately classify sentiment and intent in real-world social media conversations. This validation highlights their practical applicability for public health research. Although validation on real data strengthens confidence in the models' generalizability, future work should include testing on more diverse datasets that capture demographic, cultural, and linguistic variations. Developing multilingual models presents a promising direction, enabling the analysis of global tobacco discourse and offering valuable insights for international tobacco control efforts. Incorporating multi-user interactions and comment threads in future analyses could also enhance the models' ability to interpret user intent and sentiment in more complex social media environments.

In conclusion, while the study applies existing models, its innovation lies in the specific adaptation and fine-tuning of the Flan-T5 model for the tobacco control domain, addressing significant challenges related to data scarcity. The use of LoRA improves computational efficiency and helps avoid overfitting, ensuring the model is suitable for resource-constrained public health applications. This work demonstrates the efficacy of fine-tuned LLMs in analyzing tobacco and e-cigarette-related social media information and opens new avenues for applying artificial intelligence in public health research and practice.

## Data Availability

The datasets presented in this study can be found in online repositories. The names of the repository/repositories and accession number(s) can be found at: https://doi.org/10.7910/DVN/WQQW8S, Harvard Dataverse, V1.
